# Histological tumor response assessment in colorectal liver metastases after neoadjuvant chemotherapy: impact of the variation in tumor regression grading and peritumoral lymphocytic infiltration

**DOI:** 10.7150/jca.31493

**Published:** 2019-10-06

**Authors:** Yibo Cai, Xingang Lu, Xiu Zhu, Haixing Ju, Wenyong Sun, Wei Wu

**Affiliations:** 1 Department of Colorectal Surgery, Institute of Cancer and Basic Medicine (ICBM) of Chinese Academy of Sciences, Cancer Hospital of University of Chinese Academy of Sciences, Zhejiang Cancer Hospital, Hangzhou, China; 2 Department of Pathology, Institute of Cancer and Basic Medicine (ICBM) of Chinese Academy of Sciences, Cancer Hospital of University of Chinese Academy of Sciences, Zhejiang Cancer Hospital, Hangzhou, China

**Keywords:** Colorectal liver metastases, neoadjuvant chemotherapy, pathological response, tumor regression grading, peritumoral lymphocytic infiltration.

## Abstract

**Background:** The objective of this study was to evaluate the prognostic value of the variation in tumor regression grade (TRG) and peritumoral lymphocytic infiltration of colorectal liver metastases (CRLMs) after neoadjuvant chemotherapy (NACT).

**Methods:** A retrospective review was performed in 98 patients with CRLMs who underwent NACT between 2010 and 2016. The TRG scores and counts of TILs at the tumor-normal interface were assessed in all 176 resected liver metastases to determine their association with prognosis. According to the variation in TRG scores, 40 patients with more than one liver metastasis were divided into a decreased TRG group and a stable TRG group. An additional independent cohort of 64 patients with 106 resected liver specimens was established to validate our main findings.

**Results:** In the derivation cohort of 98 patients, 41.8% patients had a favourable pathological response to NACT (TRG 1-3), which were significantly associated with improved prognosis. Seventeen patients (42.5%) showed decreased TRG scores, and the remaining patients had stable scores. The multivariate analysis indicated that patients with decreased TRG scores had a better recurrence-free survival (RFS) compared with those with stable TRG scores (HR=0.42, *P*=0.034), and a similar trend was observed in the validation cohort (*P*=0.068). Dense TILs surrounding the metastases were present in 55.1% of the derivation cohort and associated with pathological response (*P*=0.008). Among patients with a pathological response to NACT, those with dense TILs had a superior RFS compared to those with weak TILs in both cohorts (derivation: HR=0.36, *P*=0.035; validation: HR=0.34, *P*=0.016).

**Conclusions:** Variation in TRG scores and peritumoral lymphocytic infiltration may be proposed as secondary pathological parameters to evaluate the pathological response to NACT and predict the risk of recurrence after liver surgery.

## Introduction

The liver is the most common metastatic site of colorectal cancer, and approximately 15-25% of colorectal cancer patients occur with synchronous liver metastases at diagnosis 1-3. Liver resection is the only potential curative therapeutic option and is recommended as the standard of care. Unfortunately, 60-80% of patients who undergo liver resection develop recurrence after surgery 4. Therefore, the identification of good candidates for aggressive approaches is meaningful and important. Over the past decade, effective neoadjuvant chemotherapy (NACT), including oxaliplatin- and irinotecan-based regimens, has been suggested to stratify good candidates for aggressive approaches and consolidate surgical outcomes 4-6. Additionally, a 5-point histological tumor regression grade (TRG) scoring system according to the extent of intralesional residual tumor cells and fibrosis was established to evaluate the efficacy of preoperative chemotherapy in colorectal liver metastases (CRLMs) 7. The accumulated evidence unequivocally demonstrates that major or partial pathological response to preoperative chemotherapy is significantly associated with improved survival 8-10, possibly due to the eradication of micrometastases 7, 11. The highest TRG score among multiple CRLMs has been proposed as a surrogate parameter for pathological response in most previous studies. Tanaka et al 12 found that the presence of complete pathological response to preoperative chemotherapy in at least one metastatic lesion was associated with better survival, although residual tumor cells could still be detected within the other lesions of the same patient. Therefore, the variability in TRG scores merits further attention. However, few studies have described the variation in TRG scores and its possible relationship with pathological response to NACT.

Peritumoral lymphocytic infiltration has been recognized as a reliable prognostic factor of survival outcomes in patients with colorectal cancer 13. Moreover, Okano et al assessed lymphocytic infiltration after surgical resection of the CRLMs and indicated that dense tumor infiltrating lymphocytes (TILs) presented at the tumor-normal interface (TNI) were associated with improved prognosis 14. Intra- or peritumoral lymphocytic infiltration may serve as surrogate markers of pathological response to chemotherapy. Several previous studies have demonstrated that the presence of intratumoral TILs can predict pathological response after neoadjuvant therapy in patients with breast cancer and rectal cancer 15-17. However, the correlation between the density of TILs surrounding the liver metastases and the pathological response to preoperative chemotherapy remains to be investigated in CRLMs treated with NACT.

The purposes of this study were (i) to assess the variation in TRG scores and the presence of lymphocytic infiltration surrounding the liver metastases in patients who underwent liver resection after NACT and (ii) to analyse the correlations of these two parameters with pathological response to NACT and postoperative survival.

## Materials and methods

### Patients

Consecutive patients diagnosed with synchronous or metachronous CRLMs and underwent NACT in the Zhejiang Cancer Hospital from June 2010 to June 2016 were enrolled in this study. And an independent cohort in the same institution from January 2005 to May 2010, with a longer-term follow-up, was established to validated the main findings. The study cohorts excluded patients treated with selective ablation, internal radiotherapy or hepatic arterial therapy before liver resection. Patients whose deaths were caused by postoperative complications, defined as those with life expectancy within one month after liver resection, were also excluded from enrolment. The preoperative demographics and clinicopathological data including age, gender, primary tumor location, lymph node status of the primary tumor, preoperative carcinoembryonic antigen (CEA) level, *KRAS* mutation, and presence of extrahepatic disease were collected from eligible patients. Detailed information regarding NACT and targeted therapy was also obtained. All extrahepatic metastases presented in the included patients were concomitantly resected during hepatectomy. Radiological response was assessed according to the Response Evaluation Criteria In Solid Tumors (RECIST) version 1.1 criteria 18. Follow-up was performed through regular outpatient visits or telephone interviews. Liver radiologic imaging along with the detection of serum CEA levels were regularly used to monitor tumor recurrence.

### Pathological assessment of liver metastases

**Figure 1:**
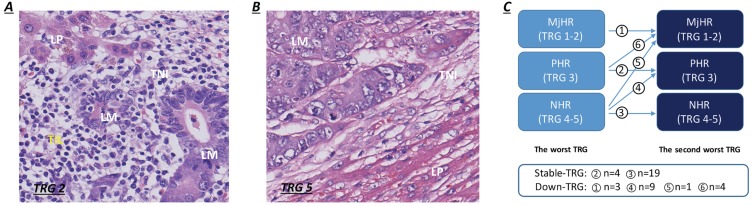
Lymphocytic infiltration at the tumor-normal interface and the pattern of variation in TRG between metastases. **A.** Dense TILs: dense lymphocytes were encountered surrounding liver metastases (>50/HPF). **B.** Weak TILs: scanty lymphocytes were observed surrounding liver metastases (≤ 50/HPF). **C.** Patients were subcategorized into two groups according to whether TRG scores of the second-worse metastases decreased or remained stable.

Informed consent for histological examination was obtained from all enrolled participants. The postoperative pathological liver resection specimens were fixed in formalin, embedded in paraffin and stained with haematoxylin-eosin (H&E). All specimens were sectioned into 5 mm thick slices. The slice revisions were performed by two experienced pathologists (W Wu and X Zhu) independently and blindly. Macroscopic assessments of the resected specimens included the number and maximum tumor diameter of metastases and the width of the resection margins. The status of liver resection margins, capsular invasion, percent intralesional necrosis, percentage of intralesional residual tumor cells and fibrosis, and degree of lymphocytic infiltration were evaluated by microscopic observations. Positive surgical margins (R1/R2) were defined as the histologic presence of residual tumor cells at or within 1 cm of the resection margins. The TRG scoring system, a 5-point scale representing pathological response, was established based upon the extent of intralesional residual tumor cells and fibrosis. TRG scores of 1 or 2 were categorized as a major histological tumor response (MjHR), a TRG score of 3 was categorized as a partial histological tumor response (PHR), and TRG scores of 4 or 5 were categorized as no histological tumor response (NHR) 7. Patients with major and partial tumor responses (TRG 1-2 and TRG 3) were merged for analysis in our study. Lymphocytic infiltration surrounding liver metastases was observed mainly at the TNI and was quantitated as the mean number of TILs per 10 high-power microscopic fields (HPFs) (400×), which were stratified as dense (> 50/HPF) or weak (≤ 50/HPF) (Fig. 1A-B) 14. For patients with multiple CRLMs, all resected lesions were evaluated using the same procedure. The pathological characteristics of liver metastases were assessed based on patient-related analyses. If the grades were different between metastases within a patient, the morphological characteristics of the worst metastasis (highest TRG score) were considered as the reference. The variability in TRG scores for multiple CRLMs was defined as a variation in TRG between the worst (highest TRG score) and second-worst metastasis (second-highest TRG score). Compared to their worst metastasis, patients were subcategorized into two groups according to whether the TRG score of the second-worse metastasis decreased (decreased TRG group, Fig. 1C) or remained stable (stable TRG group, Fig. 1C) after NACT. Patients with major pathological response in the worst metastasis were directly categorized as having a decreased TRG, on account of the sustained tumor regression exhibited in all resected specimens.

### Statistical analysis

Statistical analysis was performed using SPSS software version 19.0 (SPSS Inc., Chicago, IL). For continuous variables, the data were summarized as the mean with standard deviations (SD) or median with interquartile range (IQR) and compared using Student's t-tests or non-parametric Mann-Whitney U tests. Categorical variables were summarized as absolute values or percentages and compared using Pearson's chi-square or Fisher's exact tests. Recurrence-free survival (RFS) and overall survival (OS) were calculated from the date of liver resection to the date of recurrence or last follow-up and the date of death or last follow-up, respectively. Survival curves were estimated by the Kaplan-Meier method and compared using the log-rank test. All parameters with a P value of less than 0.1 in the univariate analysis were included in the multivariate analysis. A backward stepwise Cox proportional hazard model was fitted to identify independent predictors associated with RFS and OS after liver surgery. A two-sided *p* value of less than 0.05 was considered statistically significant.

## Results

### Clinical and histopathological characteristics in the derivation cohort

From June 2010 to June 2016, a total of 98 patients with CRLMs who underwent NACT before liver resection met the inclusion criteria. The characteristics of the patients and tumors are summarized in Table 1. The mean age at the time of liver surgery was 53.9 years, and 59.2% of patients were male. Most patients had rectal (46.9%) and advanced primary tumors (T_3-4_, 78.7%; positive lymph nodes, 56.3%). Regarding liver metastases, 66.3% of patients had synchronous metastases, 51.0% had more than one metastatic lesion and 33.7% had multiple bilobar metastases. The mean maximum diameter of the resected lesions was 34.7 mm. Positive preoperative serum CEA level was detected in more than half of the patients (63.2%). *KRAS* mutations and extrahepatic metastatic disease were observed in 26.5% and 10.2% of patients with CRLMs. Most patients (76.5%) received oxaliplatin-based NACT regimens, 15.3% received irinotecan-based regimens, 10.2% and 10.2% received cetuximab- and bevacizumab-containing regimens. The median number of NACT cycles was 4 (IQR 3-7). Microscopically, 9 patients had positive resection margins, and all of them presented with higher TRG scores (TRG 4-5). Predominant necrosis (≥50%) and liver capsule invasion occurred in 18.4% and 48.0% of the patients, respectively.

### TRG and its variation and association with NACT

The TRG of each resected CRLM was assessed after preoperative chemotherapy, and the categorization of the included patients was based upon the highest TRG score. TRG scores of 1-3 that correspond to treatment-induced tumor regression were observed in 41 patients (41.8%), whereas the remaining patients (58.2%) presented with poor tumor regression (TRG 4-5) (Table 1). The groups, stratified by TRG scores, were well-matched, except for a higher frequency of abnormal preoperative CEA level in the TRG 4-5 group (77.2% vs. 43.9%, *P*=0.001). The variation in TRG was evaluated by comparing the TRG scores (three categories: MjHR, PHR and NHR) of the worst and second-worst metastases. Nearly half of the second-worst metastatic lesions (19/40, 47.5%) showed TRG 4-5. Decreased TRG scores were observed in 17 patients (42.5%), and stable TRG scores were observed in 23 patients (57.5%, Fig. 1C). Approximately 44.0% of patients (33/75) who received oxaliplatin-based NACT presented with TRG scores of 1-3, which was 33.3% % (5/15) for irinotecan-based NACT, 50.0% (5/10) and 50.0% (5/10) for cetuximab- and bevacizumab-containing NACT regimens (Table 1). TRG 1-3 observed in 70.0% of patients with a complete or partial radiological response after NACT, whereas that for poor radiological response was 22.4% (*P*<0.001). In patients with more than one metastasis, 45.2% who received oxaliplatin-based NACT and 66.7% who received cetuximab plus chemotherapy showed decreased TRG scores (Table 4). A higher rate of TRG decline was observed in patients with CRLMs achieving complete or partial radiological response after NACT than those with no response (61.1% vs. 27.3%, *P*=0.031).

### Lymphocytic infiltration surrounding CRLMs and its association with TRG and NACT

Lymphocytic infiltration surrounding liver metastases was observed mainly at the TNI. More than half of the patients (55.1%) presented with dense TILs (>50/HPF), and the remaining patients (44.9%) presented with weak TILs (≤50/HPF) (Table 1). Lymphocytic infiltration surrounding CRLMs was relatively common in patients who achieved good pathological response (TRG 1-3: 70.7% vs. TRG 4-5: 43.8%, *P*=0.008) or complete/partial radiological response after NACT (CR+PR 80.0% vs. SD+PD 37.9%, *P*<0.001) (Table 1, 4). In terms of NACT, dense TILs were observed in 56.0% of the patients with CRLMs receiving oxaliplatin-based NACT and 70.0% receiving bevacizumab plus chemotherapy (Table 4).

### Survival analysis

**Figure 2:**
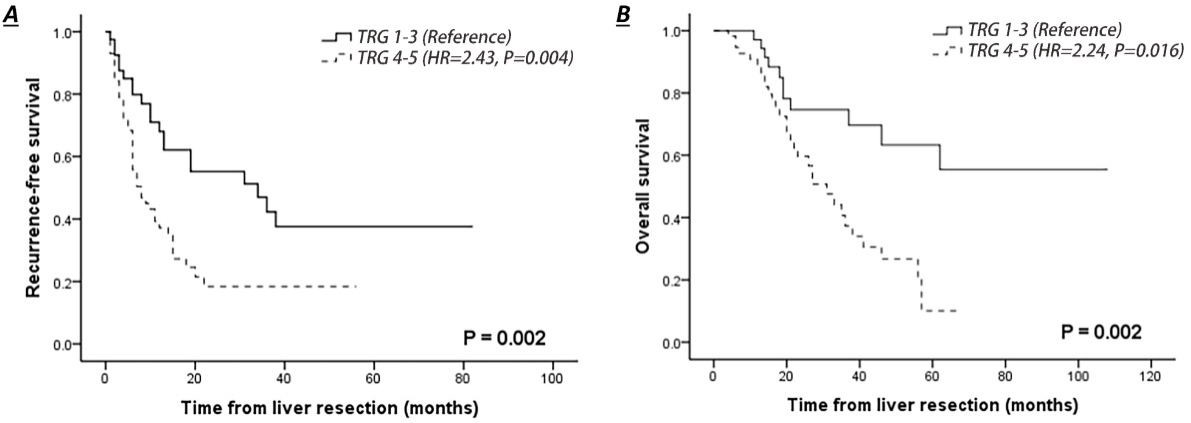
Comparison of survival curves of patients with pathological response (TRG 1-3) or no response (TRG 4-5) in liver colorectal metastasis treated by NACT. **A.** Recurrence-free survival of patients with complete clinical follow-up. **B.** Overall survival of patients with complete clinical follow-up.

The median follow-up of the entire cohort was 37 months, and one patient was lost to follow-up. The 3-year RFS and OS rates were 27.8% and 52.5%, respectively (Table 2). The survival outcome was significantly worse in patients with CRLMs who had poor pathological response to NACT (TRG 4-5 vs. TRG 1-3: RFS, hazard ratio (HR)=2.43, 95% confidence interval (CI) 1.33-4.46, *P*=0.004; OS, HR=2.24, 95% CI 1.06-4.70, *P*=0.016) (Fig. 2A-B). Patients with radiological response to NACT (CR+PR) had an improved 3-year RFS (41.8% vs. 17.6%, *P*=0.011) and a slightly higher 3-year OS rate (63.1% vs. 45.9%, *P*=0.062) than those with no response (SD+PD), but this survival improvement was not significant in a multivariate context (SD+PD vs CR+PR: RFS, HR=2.43, 95% CI 0.90-3.32, *P*=0.101; OS, HR=1.12, 95% CI 0.49-2.56, *P*=0.784).

**Figure 3:**
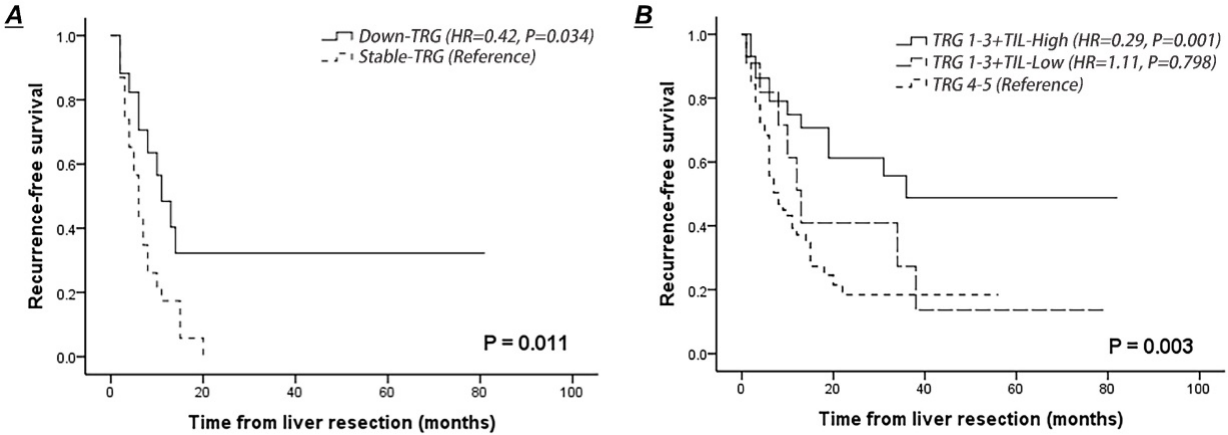
Comparison of recurrence-free survival curves of patients after NACT according to the lymphocytic infiltration at the tumor-normal interface and variation in TRG scores between metastases. **A.** Recurrence-free survival of patients presented with decreased or stable TRG scores of the second-worse metastases. **B.** Recurrence-free survival of patients presented with dense or weak TILs at the tumor-normal interface combining with histological tumor regression.

Patients with decreased TRG scores of the second-worse metastasis had a better RFS rate than those with stable scores (HR=0.42, 95% CI 0.19-0.94, *P*=0.034) (Fig. 3A). Lymphocytic infiltration surrounding liver metastasis was not associated with improved RFS in the entire cohort (*P*=0.248), whereas dense TILs surrounding liver metastasis combined with treatment-induced tumor regression (TRG 1-3) indicated a low risk of recurrence after liver resection (dense TILs: 48.7% vs. weak TILs: 27.3%; HR=0.36, 95% CI 0.14-0.93, *P*=0.035) (Fig. 3B). Notably, 12 patients (12.2%) who presented with a combination of a TRG score of 1-3 and weak TILs surrounding liver metastasis had a 3-year RFS similar to that of patients with poor pathological response (HR=0.90, *P*=0.798) (Fig. 3B).

### Validation

The validation was performed on an independent cohort of 64 consecutive patients with CRLMs and baseline variables in the validation cohort were retrospectively collected and summarized in Table S1. Exploratory analysis on the validation cohort verified that dense TILs was associated with pathological response (TRG 1-3: 65.4% vs. TRG 4-5: 26.3%, *P*=0.002) (Table S1). Patients with decreased TRG tended to have a superior RFS, although this survival difference was not statistically significant (*P*=0.068) (Figure S1). Lymphocytic infiltration around CRLM with pathological response to NACT was a significant predictor of improved recurrence-free survival (RFS) (HR=0.34, 95% CI 0.19-0.94, *P*=0.016) (Figure S1), which was consistent with the result from the derivation cohort.

## Discussion

Our study describes the histological tumor response patterns of CRLMs to preoperative chemotherapy and assesses the association of these patterns with survival in a homogeneous cohort of patients treated with NACT followed by liver resection. To date, this is the first report to focus on the variation in TRG scores across multiple liver metastases and the combination of peritumoral lymphocytic infiltration with a TRG scoring system.

Over the past decade, NACT has been widely recommended for the management of initially resectable CRLMs to identify optimal candidates for aggressive approaches by inducing tumor shrinkage that then allows for surgical removal 4-6. The assessment of tumor regression has been gradually utilized to quantify the pathological response to NACT and has served as an early parameter to predict prognosis 11, 19, 20. To date, the optimal system to effectively evaluate the histological tumor regression and capture the response of chemotherapy regimens is the semi-quantitative tumor regression grade (TRG) scoring system based on the extent of intralesional residual tumor cells and fibrosis, which was established by Rubbia-Brandt et al 7. Based on this grading system, we observed that approximately 41.8% of patients presented with a favourable score (TRG 1-3) after NACT in our derivation cohort, which was similar to the results in Western populations (44-56%) 7, 10, 21, 22. Consistent with previous results, the tumor response to NACT in the current study was strongly related to a low risk of recurrence after liver surgery 8-10, 23. Discrepancies in the tumor regression patterns in response to different chemotherapy regimens have been revealed in previous literature. Rubbia-Brandt et al reported that oxaliplatin-based regimen produced improved pathological response compared with 5-FU- or irinotecan-based regimens 7. In terms of monoclonal antibodies, bevacizumab- containing regimen provided a better pathological response than chemotherapy alone or in combination with cetuximab 10, 24. Our results showed a higher pathological response rate to NACT in patients underwent bevacizumab- or cetuximab-containing regimens (50.0%) than those with oxaliplatin- (44.0%) or irinotecan-based (33.3%) chemotherapy, although there was no significant difference (Table 1). The radiological response to NACT has been found to be corrected to the residual viable tumor burden 25. In our study, patients with a radiological response had a more favourable pathological response than those with no response (70.0% vs 22.4%, *P*<0.001). Preoperative radiological evaluation of tumor response according to RECIST criteria is essential to assess the resectability of CRLMs and affects the multidisciplinary decision-making process 8, 26, 27. Owing to the potential correlation with tumor regression and survival outcomes, radiological response to preoperative chemotherapy has been proposed as a preoperative surrogate indicator to predict pathological response before liver resection. However, this uniformity was not observed between patients with complete radiological response and complete pathologic response 12.

The variability in TRG scores between liver metastases in the same patient is always neglected in previous studies, and this study provides the first explicit approach for the assessment of variability in TRG scores. We subcategorized patients with more than one CRLM into decreased TRG and stable TRG groups, according to the variation in TRG scores between the worst (highest TRG score) and second-worst metastasis (second-highest TRG score). Discrepancies in TRG scores were observed in 42.5% of patients, which was consistent with the results from a French study (45.0%) 22 and much higher than the rate reported by Rubbia-Brandt et al (10%) 7. Selective internal radiotherapy or ablation may facilitate tumor shrinkage and influence the accurate assessment of histological tumor regression 8, 28. To eliminate interference from these additional therapies, patients treated with selective ablation, internal radiotherapy or hepatic arterial infusion chemotherapy before liver resection were excluded in our study. Although Tanaka et al did not directly describe the variability in the extent of viable tumor cells and fibrosis, they reported that 23 patients with 81 CRLMs presented with a complete pathologic response to preoperative chemotherapy, and residual tumor cells could still be detected in the remaining liver metastases (103/184, 56.0%) 12. Meanwhile, they also found that patients with at least one metastasis confirmed to achieve complete pathologic response was associated with a better survival than those with no complete pathologic response 12. These findings underline that considerable variations seem to exist in colorectal liver metastases and that its potential relationship to tumor response should not be neglected. Previous studies have confirmed that the tumor size, duration of perioperative chemotherapy, serum CEA level and preoperative chemotherapy regimens are efficient predictors of pathological response 19, 29. We speculate that tumor heterogeneity, manifesting differences in metastatic tumor burden and chemotherapy sensitivity, exists among liver metastases and results in pronounced discrepancies in TRG scores in the same patient after NACT. Our results indicated that patients with CRLMs that presented decreased TRG scores between metastases showed a significantly higher RFS rate than those with stable ones (HR=0.42, 95% CI 0.19-0.94, *P*=0.034) (Fig. 3A). All liver metastases of one patient showing pathological complete response is regarded as the optimal response to preoperative chemotherapy, and documenting the histological tumor regression of each liver lesion has been recommended by several investigators to obtain data regarding the variability in TRG scores 30. We unveil a novel insight that the variation in TRG could emerge as a supplemental method to enhance the accuracy of the classical tumor regression grading system.

Lymphocytic cells infiltrating into or around tumor nests are recognized as a histological manifestation of tumor-immune interactions. The density, composition, architecture, and function of the infiltrating lymphocytes has gradually been revealed in the past decade 31. Some investigators believe that the extent of peritumoral lymphocytic infiltration could reflect the activity of the host defence reaction and predict survival in CRLM patients underwent initial liver resection 14, 32. Indeed, it remains to be investigated whether this pathological finding is still exhibited in the liver metastases after perioperative chemotherapy. We assessed the densities of infiltrated lymphocytes at the TNI after NACT, and 55.1% of the patients were identified to have dense TILs. In the Okano et al study, compared to lymphocytic infiltration at the TNI before chemotherapy (44.0%), denser TILs were observed after NACT, which reflects the enhancement of the antitumor immune response caused by chemotherapy. This trend of elevated TIL counts is also found in rectal cancer after neoadjuvant therapy 17, 33, 34. Moreover, a strong association between dense TILs and improved RFS was described for the first time in our series for patients with TRG scores 1-3, which was not observed in the TRG 4-5 subgroup. A possible explanation for this disparity in recurrence risk between responders to NACT and nonresponders may be the differences in immune cell and immunogenicity factor composition (e.g., CD3^+^ T cells, CD8^+^ T cells, CD45RO^+^ T cells, and Trg cells), which could also give rise to the different responses to chemotherapy 35. Halama et al retrieved samples derived from liver biopsies or diagnostic excisions and quantified the TIL densities in the presumable invasive margins of liver metastases. Recursive partitioning of the densities of CD3^+^, CD8^+^, or granzyme B^+^ lymphocytes was utilized to generate a scoring system for predicting radiological response to chemotherapy 36. This predictive significance of dense TILs in the invasive margins supports the hypothesis that peritumoral lymphocytic infiltration may have an impact on the efficacy of different chemotherapy regimens and additional targeted therapy 31, 36. In vivo, 5-fluorouracil, the backbone of all chemotherapy regimens, has been demonstrated to stimulate tumor-infiltrating CD8^+^ T cells and inhibit the suppression of T cell-mediated tumor control 37. In our series, we also found a significantly higher proportion of dense TILs in patients with radiological or pathologic responses to NACT than in those with no response (*P*<0.001 or *P*=0.008). In line with this finding, we suspected that peritumoral lymphocytic infiltration after NACT could be interpreted as a simplified sign of intricate tumor-immune interaction, as well as a secondary surrogate for pathological response to NACT in patients with histological tumor regression (TRG 1-3).

Several limitations in the current study need to be recognized. Few patients in our cohort were recommended to undergo invasive detection procedures, such as pretherapeutic liver biopsies or diagnostic excision. Therefore, the baseline counts of TILs and changes in the density of infiltrating lymphocytes during preoperative chemotherapy could not be assessed in this retrospective study. Owing to the potential complications derived from invasive detections and the merits of imaging-based tumor response assessments in the candidate selection for aggressive approaches, we do not support the application of pretherapeutic liver biopsies or diagnostic excision exclusively to predict response to NACT. Another definite limitation is that the type and composition of TILs at the TNI were not separately characterized by gene expression profiling and immunohistochemistry. The relationship between distinct TILs surrounding liver metastases and prognosis merits more exploration. We performed simple approaches to quantify the variation in TRG scores and lymphocytic infiltration at the TNI that are likely to be performed and promoted in clinical practice. Finally, the sample sizes in subgroups stratified by NACT regimens are too small to reach any definitive conclusions or recommendations, on account of the inadequate statistical power. Although our results demonstrated the clinical potential of variations in TRG scores and lymphocytic infiltration at the TNI as prognostic biomarkers, further prospective and large-scale external validations of the scoring scale are required.

In conclusion, our study validates the strong association between histological tumor regression and favourable prognosis in patients who underwent liver resection after NACT. Histological TRG scores could be proposed as a surrogate marker of pathological response. Then, we further developed a feasible and effective approach to assess the variability in TRG scores between liver metastases in the same patient. A decreased TRG score and increased TIL concentration at the TNI are confirmed to be related to favourable tumor response to NACT and prolonged RFS after liver surgery, which may be regarded as secondary pathological parameters of the histological tumor response assessment.

## Supplementary Material

Supplementary figures and tables.

## Figures and Tables

**Table 1:** Clinicopathological characteristics of colorectal cancer with liver metastases undergoing liver resection followed by preoperative chemotherapy.

Variables	Overall (n=98)	TRG 1-3 (n=41)	TRG 4-5 (n=57)	P
Age, mean (SD), years	53.9 (12.4)	54.9 (13.2)	53.1 (11.9)	0.497^†^
**Gender**				0.470^‡^
Male	58 (59.2 %)	26 (63.4 %)	32 (56.1 %)	
Female	40 (40.8 %)	15 (36.6 %)	25 (43.9 %)	
**Location of primary tumor**				0.654^‡^
Right-sided	26 (26.5 %)	12 (29.3 %)	14 (24.6 %)	
Left-sided	26 (26.5 %)	12 (29.3 %)	14 (24.6 %)	
Rectum	46 (47.0 %)	17 (41.4 %)	29 (50.8 %)	
**Pathologic stage T_3-4_^#^**	74/94 (78.7 %)	31/40 (77.5 %)	43/54 (79.6 %)	0.796^‡^
**Lymph nodes metastasis^ #^**	53/94 (56.3 %)	21/40 (52.5 %)	32/54 (59.3 %)	0.514^‡^
**Synchronous CRLMs**	65 (66.3 %)	29 (70.7 %)	36 (63.2 %)	0.434^‡^
**Maximum diameter of CRLMs,** **mean (SD), mm**	34.7 (26.1)	27.5 (25.0)	39.9 (25.8)	0.130^†^
**Number of CRLMs ≥ 2**	50 (51.0 %)	17 (41.5 %)	33 (57.9 %)	0.108^‡^
**Bilobar CRLMs**	33 (33.7 %)	14 (34.1 %)	19 (33.3 %)	0.933^‡^
**Positive Pre-operative CEA**	62 (63.2 %)	18 (43.9 %)	44 (77.2 %)	0.001^‡^
**Mutated *KRAS***	26 (26.5 %)	10 (24.3 %)	16 (28.1 %)	0.684^‡^
**Extrahepatic disease**	10 (10.2 %)	4 (9.8 %)	6 (10.5 %)	0.901^§^
**Backbone of NACT**				0.722^§^
Oxaliplatin	75 (76.5 %)	33 (80.5 %)	42 (73.6 %)	
Irinotecan	15 (15.3 %)	5 (12.2 %)	10 (17.5 %)	
Oxaliplatin+irinotecan	8 (8.2 %)	3 (7.3 %)	5 (8.8 %)	
**Targeted therapy**				0.709^‡^
Cetuximab	10 (10.2 %)	5 (12.2 %)	5 (8.8 %)	
Bevacizumab	10 (10.2 %)	5 (12.2 %)	5 (8.8 %)	
Without	78 (79.6 %)	31 (75.6 %)	47 (82.4 %)	
**Preoperative chemotherapy > 1^st^ line**	9 (9.2 %)	3 (7.3 %)	6 (10.5 %)	-^*^
**Number of cycles, median (IQR)**	4 (3-7)	4 (3-6)	5 (3-8)	0.214^¶^
**Radiological response**				<0.001^‡^
Response (CR+PR)	40 (40.8 %)	28 (68.3 %)	12 (21.1 %)	
No response (SD+PD)	58 (59.2 %)	13 (31.7 %)	45 (78.9 %)	
**R1/2 margin**	9 (9.2 %)	0 (0.0 %)	9 (15.8 %)	-^*^
**Necrosis ≥ 50%**	18 (18.4 %)	8 (19.5 %)	10 (17.5 %)	0.804^‡^
**Capsular invasion**	47 (48.0 %)	14 (34.1 %)	33 (57.9 %)	0.144^‡^
**Dense-TILs**	54 (55.1 %)	29 (70.7 %)	25 (43.8 %)	0.008^‡^
**Second-worst TRG** ^##^				-^*^
1-3	21/40 (52.5 %)	11/11 (100.0 %)	10/29 (34.5 %)	
4-5	19/40 (47.5 %)	0/11 (0.0 %)	19/29 (65.5 %)	
**Variability of TRG** ^##^				0.153^§^
Decreased TRG	17/40 (42.5 %)	7/11 (63.6 %)	10/29 (34.5 %)	
Stable TRG	23/40 (57.5 %)	4/11 (36.4 %)	19/29 (65.5 %)	

**Abbreviations:** TRG: tumor regression grade; SD: standard deviation; CRLMs: colorectal liver metastases; CEA: carcinoembryonic antigen; NACT: neoadjuvant chemotherapy; IQR: interquartile range; CR: complete response; PR: partial response; SD: stable disease; PD: progressive disease; TILs: tumor infiltrating lymphocytes. ^#^Pathologic information of the primary tumor was missing for 4 patients. ^##^There are 40 patients with multiple CRLMs receiving the evaluation of variability of TRG. ^†^Student's t test. ^‡^Pearson Chi-square test. ^§^ Fisher's exact test. ^*^ The statistical analysis was not performed on account of the small sample sizes in subgroup. ^¶^Wilcoxon rank-sum test.

**Table 2:** Potential predictors for recurrence-free survival and overall survival in colorectal cancer with liver metastasis undergoing liver resection followed by preoperative chemotherapy.

Variable (n=97) *	Recurrence-free survival				Overall survival			
	Univariate		Multivariate ^†^		Univariate		Multivariate ^†^	
	3-year RFS	P	HR (95% CI)	P	3-year OS	P	HR (95% CI)	P
Overall	27.8 %				52.5 %			
Lymph nodes status ^#^								
Negative (n=40)	47.6 %	0.006	1 (Reference)		65.9 %	0.020	1 (Reference)	
Positive (n=53)	14.7 %		1.85 (1.06-3.22)	0.030	44.1 %		2.47 (1.22-5.02)	0.012
Number of metastases								
1 (n=47)	45.0 %	<0.001	1 (Reference)		60.6 %	0.037	1 (Reference)	
≥ 2 (n=50)	11.5 %		1.85 (1.06-3.24)	0.030	43.6 %		1.40 (0.69-2.85)	0.354
*KRAS*								
Wild (n=71)	28.9 %	0.103	-		58.4 %	0.091	1 (Reference)	
Mutated (n=26)	23.9 %		-		35.8 %		2.04 (1.01-4.14)	0.047
TRG								
1-3 (n=40)	42.3 %	0.002	1 (Reference)		74.6 %	0.002	1 (Reference)	
4-5 (n=57)	18.4 %		2.43 (1.33-4.46)	0.004	37.3 %		2.24 (1.06-4.70)	0.016

Abbreviations: RFS: recurrence-free survival; OS: overall survival; HR: hazard ratio; CI: confidence interval; TRG: tumor regression grade. *Survival data is from 97 patients and 1 patient was lost of follow-up. † HR was adjusted by the lymph modes status, number of metastases, targeted therapy, radiological response, TRG, bilobar liver metastases, resection margin in multivariate analyses of RFS and adjusted by the lymph modes status, number of metastases, KRAS status, radiological response, TRG in OS. #Pathologic information of the primary tumor was missing for 4 patients and 1 patient was lost of follow-up.

**Table 3:** Additional histopathological predictors for recurrence-free survival and overall survival in patients with different TRG undergoing liver resection after preoperative chemotherapy.

Variable (n=97) *	Recurrence-free survival		Overall survival	
	3-year RFS/Adjusted HR^†^ P		3-year OS/Adjusted HR^†^ P	
**Second-worst TRG ^#^**				
1-3 (n=21)	1(Reference)		57.6 %	0.791
4-5 (n=19)	1.35 (0.58-3.10)	0.485	46.9 %	
**Variability of TRG ^#^**				
Stable (n=17)	1(Reference)		49.0 %	0.577
Decreased (n=23)	0.42 (0.19-0.94)	0.034	56.6 %	
**Necrosis**				
<50% (n=79)	27.6 %	0.434	56.1 %	0.272
≥ 50% (n=18)	27.8 %		43.8 %	
**TILs**				
Weak (n=43)	24.9 %	0.248	55.0 %	0.833
Dense (n=54)	29.9 %		52.5 %	
**Capsule invasion**				
No (n=50)	32.6 %	0.353	59.8 %	0.416
Yes (n=47)	23.2 %		47.6 %	

**Abbreviations:** TRG: tumor regression grade; RFS: recurrence-free survival; OS: overall survival; HR: hazard ratio; TILs: tumor infiltrating lymphocytes; NA: not acceptable.^*^Survival data is from 97 patients and one patient was lost of follow up. ^†^HR was adjusted by the lymph node status, number of metastases, targeted therapy, TRG in multivariate analyses of RFS and adjusted by the lymph modes status, *KRAS* status, TRG in OS. ^#^There are 40 patients with multiple CRLMs receiving the evaluation of variability of TRG.

**Table 4:** Information about NACT in patients stratified by the variability of TRG and lymphocytic infiltration.

Variables	Variability of TRG (n=40)		TILs (n=98)	
	Decreased (n=17)	Stable (n=23)	Dense (n=54)	Weak (n=44)
**Backbone of NACT**				
Oxaliplatin	14 (45.1 %)	17 (54.9 %)	42 (56.0 %)	33 (44.0 %)
Irinotecan	3 (50.0 %)	3 (50.0 %)	9 (60.0 %)	6 (40.0 %)
Oxaliplatin+irinotecan	0 (0.0 %)	3 (100.0 %)	3 (37.5 %)	5 (62.5 %)
**Targeted therapy**				
Cetuximab	4 (66.7 %)	2 (33.3 %)	6 (60.0 %)	4 (40.0 %)
Bevacizumab	2 (40.0 %)	3 (60.0 %)	7 (70.0 %)	3 (30.0 %)
Without	11 (37.9 %)	18 (62.1%)	41 (52.6)	37 (47.4%)
**Preoperative chemotherapy > 1^st^ line**	3 (75.0 %)	1 (25.0 %)	6 (50.0 %)	6 (50.0 %)
**Number of cycles, median**	6	4	5	4
**Radiological response**				
Response (CR+PR)	11 (61.1 %)	7 (38.9 %)	32 (80.0 %)	8 (20.0 %)
No response (SD+PD)	6 (27.3 %)	16 (72.7 %)	22 (37.9 %)	36 (62.1 %)
P value		0.031^†^		<0.001^†^

**Abbreviations:** TRG, Tumor regression grade; TILs, Tumor infiltrating lymphocytes; NACT, Neoadjuvant chemotherapy; CR, Complete response; PR, Partial response; SD, Stable disease; PD, Progressive disease. ^†^ Pearson Chi-square test.
