# Supplementary material for: Histological tumor response assessment in colorectal liver metastases after neoadjuvant chemotherapy: impact of the variation in tumor regression grading and peritumoral lymphocytic infiltration
Source: J Cancer. 2019 Oct 6;10(23):5852–61. doi: 10.7150/jca.31493 (PMC6843876; doi:10.7150/jca.31493)
Supplement: Supplementary file 1 — Supplementary figures and tables. [file jcav10p5852s1.pdf]

Supplementary materials

Table S1. Clinicopathological characteristics of colorectal liver metastases undergoing liver resection followed by preoperative chemotherapy in the validation cohort

Variables	Overall (n=64)	TRG 1-3 (n=26)	TRG 4-5 (n=38)	P
**Age, mean (SD), years**	55.8 (8.9)	56.8 (8.5)	55.1 (9.3)	0.456†
**Male gender**				0.100‡
Male	39 (60.9 %)	19 (73.1 %)	20 (52.6 %)	
Female	25 (39.1 %)	7 (26.9 %)	18 (47.4 %)	
**Location of primary tumor**				0.217‡
Right-sided	20 (31.3 %)	11 (42.3 %)	9 (23.7 %)	
Left-sided	12 (18.7 %)	3 (11.5 %)	9 (23.7 %)	
Rectum	32 (50.0 %)	12 (46.2 %)	20 (52.6 %)	
**Pathologic stage T_{3-4}**	60 (93.8 %)	24 (92.3 %)	36 (94.7 %)	1.000§
**Lymph nodes metastasis**	50 (78.1 %)	18 (69.2 %)	32 (84.2 %)	0.155‡
**Synchronous CRLMs**	27 (42.2 %)	11 (42.3 %)	16 (42.1 %)	0.987‡
**Maximum diameter of CRLMs, mean (SD), mm**	36.7 (21.3)	35.9 (20.2)	37.9 (23.1)	0.720†
**Number of CRLMs ≥ 2**	38 (59.3 %)	13 (50.0 %)	25 (65.7 %)	0.207‡
**Bilobar CRLMs**	14 (21.9 %)	4 (15.4 %)	10 (26.3 %)	0.367§
**Positive pre-operative CEA**	43 (67.2 %)	12 (46.2 %)	31 (81.6 %)	0.003‡
**Mutated *KRAS*** [#]	8/43 (18.6 %)	5/19 (26.3 %)	3/24 (12.5 %)	0.362§
**Extrahepatic disease**	4 (6.2 %)	2 (7.7 %)	2 (5.3 %)	1.000§
**Backbone of NACT**				-*
Oxaliplatin	50 (78.1 %)	21 (80.8 %)	29 (76.3 %)	
Irinotecan	9 (14.1 %)	4 (15.4 %)	5 (13.2 %)	
Oxaliplatin+irinotecan	5 (7.8 %)	1 (3.8 %)	4 (10.5 %)	
**Targeted therapy**				-
Cetuximab	4 (6.3 %)	1 (3.8 %)	3 (7.9 %)	
Bevacizumab	3 (4.7 %)	2 (7.7 %)	1 (2.6 %)	
Without	57 (89.0 %)	23 (88.5 %)	34 (89.5 %)	
**Preoperative chemotherapy > 1st line**	10 (15.6 %)	3 (11.5 %)	7 (18.4 %)	0.514§
**Number of cycles, median (IQR)**	4 (2-6)	4 (2-6)	4 (2-5)	0.771¶
**Radiological response**				0.006‡
Response (CR+PR)	24 (37.5 %)	15 (57.7 %)	9 (23.7 %)	
No response (SD+PD)	40 (62.5 %)	11 (42.3 %)	29 (76.3 %)	
**R1/2 margin**	3 (4.7 %)	0 (0.0 %)	3 (7.9 %)	-*
**Capsular invasion**	40 (62.5 %)	12 (46.2 %)	28 (73.7 %)	0.025‡
**Dense-TILs**	26 (40.6 %)	17 (65.4 %)	10 (26.3 %)	0.002‡
**Second-worst TRG** [#]				-*
1-3	12/25 (48.0 %)	3/3 (100.0 %)	9/22 (40.9 %)	
4-5	13/25 (52.0 %)	0/3 (0.0 %)	13/22 (59.1 %)	

**Variability of TRG**[#]				-[*]
Decreased TRG	12/25 (48.0 %)	3/3 (100.0 %)	9/22 (40.9 %)	
Stable TRG	13/25 (52.0 %)	0/3 (0.0 %)	13/22 (59.1 %)	

Abbreviation: TRG, tumor regression grade; SD, standard deviation; CEA, carcinoembryonic antigen; NACT, neoadjuvant chemotherapy; IQR, interquartile range; CR, complete response; PR, partial response; SD, stable disease; PD, progressive disease; TILs, tumor infiltrating lymphocytes.

[#] There are 25 patients with multiple CRLMs receiving the evaluation of variability of TRG.

[†] Student's t test.

[‡] Pearson Chi-square test.

[§] Fisher's exact test.

[*] The sample size was too small for valid statistical analysis.

[¶] Wilcoxon rank-sum test.

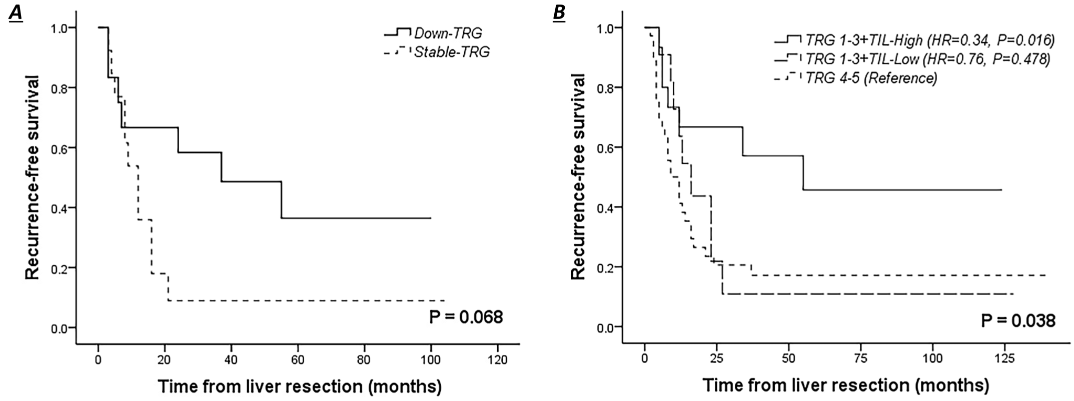

Fig. S1 Comparison of recurrence-free survival curves in the validation cohort according to peritumoral lymphocytic infiltration and variation in TRG scores between metastases. ***A.*** Recurrence-free survival of patients presented with decreased or stable TRG scores of the second-worse metastases. ***B.*** Recurrence-free survival of patients presented with dense or weak TILs at the tumor–normal interface combining with histological tumor regression.
